# Nursing care of patients with acute suppurative appendicitis and perforation in mid pregnancy: A case report

**DOI:** 10.1097/MD.0000000000043556

**Published:** 2025-07-25

**Authors:** Bin Sun, Na Zhang, Xiuying He

**Affiliations:** a Department of the International Special Needs Ward, First Hospital of China Medical University, Shenyang, Liaoning Province, China; b Department of Hepatobiliary Surgery, First Hospital of China Medical University, Shenyang, Liaoning Province, China.

**Keywords:** acute suppurative appendicitis with perforation, appendectomy, mid pregnancy, perioperative care

## Abstract

**Rationale:**

Pregnancy complicated with appendicitis is a common and serious disease, with atypical clinical manifestations and easy confusion of diseases. The prognosis is mainly related to early diagnosis and timely surgical treatment, as well as the gestational age.

**Patient concerns:**

This is a patient in mid pregnancy who experienced abdominal pain, high fever for several days, and examination reports indicating acute appendicitis. Due to congestion of pelvic organs and appendix during pregnancy, inflammation developed rapidly, resulting in suppuration and perforation of the appendix, requiring emergency surgery.

**Diagnoses:**

The final diagnosis is mid pregnancy, acute suppurative appendiceal perforation.

**Interventions:**

Due to the need to alleviate the patient’s current symptoms and protect the fetus during pregnancy, emergency surgery is required. During the surgery, the patient’s appendix was found to have suppurative perforation and severe infection. After the surgery, a large amount or combination of antibiotics is needed, but the impact of the drugs on the fetus should be considered. Therefore, anti-infection, nutritional support, and fetal protection treatment should be carried out simultaneously.

**Outcomes:**

The patient’s infection decreased, fever symptoms disappeared and there was no abdominal pain after eating. The fetus was not affected by the disease and was discharged smoothly.

**Lessons:**

For pregnant patients, both the patient, and the fetus should be treated and protected simultaneously. By studying this case, we aim to enhance the clinical thinking of nursing staff and improve the quality of nursing service technology.

## 1. Introduction

Acute appendicitis (AA) can occur in various age groups and populations, often occurring in young adults, with a prevalence of 20 to 30 years old. It is more common in males than females.^[1]^ Acute purulent appendicitis often develops from acute simple appendicitis, with obvious swelling of the appendix, highly congested serosa, and purulent exudate covering the surface, also known as acute cellulitis appendicitis. Under the microscope, the ulcerated surface of the appendix mucosa is enlarged and extends deep to the muscular and serosal layers, with small abscesses in each layer and pus accumulation in the cavity.^[2]^ There is thin pus in the abdominal cavity around the appendix, forming localized peritonitis. Perforation often occurs on the opposite side of the mesenteric margin at the base and proximal end of the appendix; if the perforation is not encapsulated and the infection spreads, it can cause acute diffuse peritonitis.^[3]^ Appendicitis is the most common non obstetric complication during pregnancy, with an incidence rate of 1 case per 500 to 2000 pregnancies. However, appendicitis during pregnancy is also more prone to complications.^[4]^ The most noteworthy thing is that pregnant women have a higher risk of developing appendiceal perforation, with 43% in pregnant women and 4% to 19% in the general population. Perforation carries a serious risk of intraperitoneal infection and the incidence of maternal, and fetal death is higher.^[5]^ Laparoscopic appendectomy (LA) is the standard treatment for AA in the general population. However, the safety of LA during pregnancy remains a controversial issue. Studies have shown that compared to open appendectomy, LA during pregnancy has a significantly higher rate of fetal loss in LA. The preterm birth rate after laparoscopic approach seems similar or slightly better. For pregnant women who require surgical intervention, open appendectomy seems to be a safer option.^[6]^ There are also studies showing that LA for the treatment of AA is significantly associated with shortened surgical time and hospital stay. The obstetric outcomes of open and LA cohorts are comparable, and the research results support laparoscopic treatment for AA during pregnancy.^[5]^ Regardless of which surgical treatment method is chosen, the requirements for the surgeon’s judgment, operational skills, and postoperative care are extremely high. To ensure the dual safety of the patient and the fetus, we now share the nursing care of a patient with acute suppurative appendicitis and perforation in mid pregnancy who underwent appendectomy. The patient has recovered and been discharged from the hospital, and both the patient and the fetus have developed well during follow-up.

## 2. Clinical data

### 2.1. General information

Patient, female, 34 years old, complained of abdominal pain 2 days ago without any obvious cause. She sought medical attention at an external hospital for ultrasound examination, which revealed a mass on the right side of the umbilical cord with surrounding exudation, suggesting AA; i received symptomatic treatment with intravenous antibiotics in an external hospital, but my abdominal pain did not improve. I am now seeking further treatment at our hospital. Admission examination: body temperature 37.8℃, pulse 106 times/min, respiration 18 times/min, blood pressure 129/80 mm Hg. Clear and articulate, with no yellow staining on the skin and sclera, no palpable enlarged superficial lymph nodes, flat abdomen, tenderness in the lower right abdomen (+), accompanied by rebound pain (+) and muscle tension (+), small liver and spleen, and no palpable subcostal pain. Mobile voiced sounds (−), normal bowel sounds, 3 beats/min. There is no edema in the lower limbs. Previously, left knee ligament reconstruction was performed in 2014. The results of the emergency blood test upon admission showed abnormal levels of 19.73 × 10^9^/L white blood cells, 18.19 × 10^9^/L granulocytes, and 1.420 ng/mL procalcitonin, prothrombin time 13.8 second, Fibrinogen 7.03 g/L, d-dimer 2.51 μg/mL, potassium ions are 3.2 mmol/L, sodium ions are 133.5 mmol/L, and C-reactive protein 269 mg/L.

### 2.2. Treatment and outcome

On May 9, 2024 at 16:25, the patient underwent appendectomy in the emergency department under lumbar epidural block anesthesia (referred to as double block anesthesia) on the day of admission. The surgeon made a vertical incision of about 3 cm between the navel and the midpoint of the right pubic symphysis, sequentially cutting through the skin, subcutaneous tissue, and the fascia of the external oblique muscle. Bluntly separated the internal oblique muscle and the transverse abdominal muscle, lifted and cut open the peritoneum, and placed a protective ring on the incision. Exploration revealed the accumulation of purulent fluid in the abdominal cavity, which was aspirated thoroughly. The ileocecal region was found along the colon band, and the appendix was found downwards. The appendix was located in the anterior position of the intestine, with obvious edema and a length of about 6 cm and a diameter of about 1 cm. The surface was covered with pus coating, and some showed perforation. The root of the appendix adhered to the surrounding intestinal wall group, and the surrounding intestinal wall showed obvious edema. Sequential appendectomy. The total duration of the surgery was 57 minutes. After completion, the patient returned to the ward at 18:41 and was given electrocardiogram monitoring, oxygen therapy, anti-inflammatory, symptomatic, and fetal protection treatment.

## 3. Nursing

### 3.1. Fever care

Elevated body temperature: Related to factors such as appendicitis, suppuration, perforation, and abdominal infection in patients.

At the time of admission, the patient’s body temperature reached 37.9℃. As she was in mid pregnancy, no medication was used to cool her down. An emergency blood routine showed 19.73 × 10^9^/L of white blood cells, 18.19 × 10^9^/L of granulocytes, 1.420 ng/mL of procalcitonin, and 269 mg/L of C-reactive protein. Inflammatory markers were significantly elevated. Cefoperazone sodium and sulbactam sodium stock solution were tested for sensitivity, and the skin test result was negative. The patient was given intravenous infusion of 0.9% sodium chloride injection 250 mL + Cefoperazone sodium and sulbactam sodium 3.0 g twice a day on the first day after surgery, with an interval of more than 6 hours between surgery and postoperative treatment. The patient fasted with water and assisted in physical cooling by wiping with warm water for cooling treatment. On the first day after surgery, follow-up blood routine showed 13.68 × 10^9^/L white blood cells, 12.53 × 10^9^/L granulocytes, 1.190 ng/mL procalcitonin, and 257 mg/L C-reactive protein. On the second day after surgery, blood routine examination showed white blood cells at 10.50 × 10^9^/L, granulocytes at 9.17 × 10^9^/L, procalcitonin at 0.869 ng/mL, and C-reactive protein at 150 mg/L. On the fifth day after surgery, follow-up blood routine showed 7.65 × 10^9^/L white blood cells, 5.81 × 10^9^/L granulocytes, 0.197 ng/mL procalcitonin, and 12.00 mg/L C-reactive protein. All inflammatory indicators decrease day by day, and the patient has no fever symptoms. Sepsis of the appendix is a clinical manifestation of abdominal infection, mostly caused by Gram bacteria and accompanied by a certain amount of mixed infections of anaerobic bacteria. Some patients may also have fungal infections, with Escherichia coli as the main bacterium.^[7]^ The initial empirical anti infection treatment for mild to moderate abdominal infections should cover nonresistant Enterobacteriaceae bacteria and anaerobic bacteria. Based on relevant guidelines, consensus, and combined with the patient’s own situation, postoperative monotherapy for this patient can choose enzyme inhibitor complex preparations (such as cefoperazone sulbactam, ertapenem, etc).^[8,9]^ The patient’s postoperative temperature was normal, the surgical incision healed well, without redness, swelling, or exudation.

### 3.2. Dietary care

Nutrition is lower than the body’s requirements: related to surgical fasting, pregnancy, and infection factors.

The patient, due to the concurrent suppuration and perforation of appendicitis, was prevented from exacerbating infection and peritonitis. Therefore, on the day of surgery, they began to fast water before surgery and switched to a full fluid diet on the third day after surgery. During the fasting period, in order to supplement the nutritional needs of the body and fetal growth, patients closely monitor blood outcome monitoring indicators, receive fluid replacement therapy to maintain electrolyte balance, and receive total parenteral nutrition to supplement calories and nutritional support,^[10,11]^ 10% glucose injection 1000 mL + 0.9% sodium chloride injection 1000 mL + 10% potassium chloride injection 30 mL + 10% calcium gluconate 10 mL, intravenous infusion once a day. According to the patient’s condition, reduce the amount of venous fluid daily and increase the amount of oral fluid.

### 3.3. Fetal care

The danger of fetal injury: related to factors such as inflammation, infection, and suppuration.

The patient is in early pregnancy with acute suppurative appendicitis accompanied by appendiceal perforation, severe infection, elevated body temperature, and should not abuse drugs to avoid increasing fetal risk. Emergency surgical treatment was performed to alleviate the patient’s abdominal pain and infection symptoms, and reduce the potential danger to the fetus. During and after surgery, patients are given real time monitoring of electrocardiogram, blood pressure, and oxygen saturation, as well as continuous moderate flow oxygen inhalation, to closely monitor changes in the patient’s vital signs. Please consult with the obstetrics department and provide the patient with 500 mL of 5% glucose injection and 10 g of magnesium sulfate injection, intravenous infusion once a day, for fetal protection treatment. Magnesium sulfate belongs to the category of calcium ion antagonists and is often used as a contraction inhibitor in clinical practice. Magnesium ions, as the main component, can compete with calcium ions in the body, inhibit the central nervous system of patients, promote skeletal muscle relaxation, and also produce anti spastic and sedative effects, metabolizing proteins and carbohydrates.^[12]^ In addition, magnesium sulfate contains magnesium ions that can inhibit the production of acetylcholine in motor nerve endings, promote nerve and muscle conduction to be blocked, and act on uterine muscle cells.^[13]^

### 3.4. Rehabilitation nursing

The risk of venous thrombosis: Related to factors such as high blood coagulation and limited mobility during pregnancy in patients.

The patient’s pregnancy is influenced by hormone levels, which increase the estrogen levels in the pregnant woman’s body, stimulating the secretion of platelets, coagulation factors, plasminogen activator inhibitors, and other substances in the blood, leading to an increase in blood coagulation.^[14]^These physiological changes will lead to a total incidence of venous thromboembolism of approximately 1 to 2/1000 pregnancies in general pregnant women, with a pregnancy risk increased by 5 to 10 times compared to age matched non-pregnant peers.^[15]^ Therefore, for pregnant women with high venous thromboembolism scores, nursing staff should strengthen relevant health education for patients, carry out basic prevention, allow early mobilization of the patient’s condition, adopt the principle of “on the bed → beside the bed → indoor → outdoor,” and gradually increase the amount of activity. Active and passive movements of both lower limbs, ankle pump movements, with 10 groups per movement per hour.^[16]^

### 3.5. Care for abdominal distension

Abdominal distension: related to factors such as pregnancy and postoperative non defecation.

On the second day after surgery, the patient complained of abdominal distension, lack of defecation, abdominal tenderness, no muscle tension, and abdominal protrusion. Ten milliliters of Kaiseru was administered via the anus. The patient expelled 50 mL of yellow watery stool and reported relief of abdominal distension.

### 3.6. Psychological care

Anxiety: Related to factors such as patients undergoing surgical treatment, concerns about prognosis and the fetus, and lack of knowledge.

The patient is a first-time pregnancy, and when faced with surgical treatment, they experience certain psychological worries and concerns, leading to increased psychological burden and anxiety. Medical staff should strengthen the psychological care of patients to prevent worsening anxiety, poor cooperation, and delayed treatment. During this period, doctors provide patients and their families with information on their condition and risks before surgery, allowing them to fully understand the surgical process and disease-related knowledge, answer questions, and alleviate their concerns. Nurses provide patients with sufficient preoperative preparation, listen to their concerns, provide timely psychological comfort, and help them relax and have peace of mind during surgery. Medical staff use their professional knowledge to gain full trust from patients and successfully complete the surgery. In addition, pay attention to the postoperative emotional changes of patients, and also strengthen their psychological care to reduce their psychological burden. Psychological care runs through the entire hospitalization period of patients.

## 4. Conclusion

AA is the most common non obstetric cause of abdominal pain in pregnancy. The incidence rate is extremely high. Pregnant patients may show atypical examination, which makes rapid progress and brings difficulties to diagnosis and treatment, thus bringing challenges to clinicians.^[17]^ Pregnant appendicitis is associated with adverse pregnancy outcomes,^[18]^ including fetal loss, premature birth, perinatal morbidity and mortality. Therefore, timely treatment and accurate diagnosis are crucial and highly professional tasks. On the basis of interdisciplinary collaboration, it is necessary to develop planned diagnosis, treatment, and nursing measures based on the characteristics of the patient’s pregnancy. Early intervention and precise nursing are crucial for the patient’s recovery. Through this case, describe the treatment and nursing process, share accurate treatment methods and nursing measures, provide patients with precise medical care and high-quality care, and ensure the safety of patients and fetuses.

## Author contributions

**Conceptualization:** Bin Sun, Na Zhang.

**Data curation:** Bin Sun, Xiuying He.

**Formal analysis:** Bin Sun.

**Funding acquisition:** Na Zhang, Xiuying He.

**Methodology:** Na Zhang.

**Resources:** Na Zhang, Xiuying He.

**Supervision:** Xiuying He.

**Writing – original draft:** Bin Sun.

**Writing – review & editing:** Xiuying He.
